# The MINDMAP project: mental well-being in urban environments: Design and first results of a survey on healthcare planning policies, strategies and programmes that address mental health promotion and mental disorder prevention for older people in Europe

**DOI:** 10.1007/s00391-017-1290-7

**Published:** 2017-08-17

**Authors:** L. Neumann, U. Dapp, W. Jacobsen, F. van Lenthe, W. von Renteln-Kruse

**Affiliations:** 1 Geriatrics Centre, Scientific Department at the University of Hamburg, Albertinen-Haus, Sellhopsweg 18-22, 22459 Hamburg, Germany; 2 Department of Public Health, Erasmus University Rotterdam, Rotterdam, The Netherlands

**Keywords:** Mental health, Geriatrics, Urban environment, Longitudinal cohort ageing studies, Functional competence, Mentale Gesundheit, Geriatrie, Urbane Umwelt, Langzeit-Kohorten-Altersstudien, Funktionale Kompetenz

## Abstract

**Background:**

The MINDMAP consortium (2016–2019) aims to identify opportunities provided by the urban environment for the promotion of mental well-being and functioning of older people in Europe by bringing together European cities with urban longitudinal ageing studies: GLOBE, HAPIEE, HUNT, LASA, LUCAS, RECORD, Rotterdam Study, Turin Study. A survey on mental healthcare planning policies and programmes dedicated to older persons covering the range from health promotion to need of nursing care was performed for profound data interpretation in Amsterdam, Eindhoven, Hamburg, Helsinki, Kaunas, Krakow, London, Nord-Trøndelag, Paris, Prague, Rotterdam and Turin.

**Objectives:**

To collect detailed information on healthcare planning policies and programmes across these European cities to evaluate variations and to delineate recommendations for sciences, policies and planners using experience from evidence-based practice feedback from the MINDMAP cities.

**Materials and methods:**

The MINDMAP partners identified experts in the 12 cities with the best background knowledge of the mental health sector. After pretesting, semi-structured telephone interviews (1–2 h) were performed always by the same person. A structured evaluation matrix based on the geriatric functioning continuum and the World Health Organization (WHO) Public Health Framework for Healthy Ageing was applied.

**Results:**

A complete survey (12 out of 12) was performed reporting on 41 policies and 280 programmes on the city level. It appeared from extensive analyses that the focus on older citizens, specific target groups, and multidimensional programmes could be intensified.

**Conclusion:**

There is a broad variety to cope with the challenges of ageing in health, and to address both physical and mental capacities in older individuals and their dynamic interactions in urban environments.

## Zusammenfassung

**Hintergrund:**

Das MINDMAP Konsortium (2016–2019) erforscht Zusammenhänge zwischen städtischer Umwelt und Lebensqualität zur Förderung mentaler und funktionaler Gesundheit älterer Menschen in Europa durch die Zusammenführung urbaner Langzeit-Kohorten: GLOBE, HAPIEE, HUNT, LASA, LUCAS, RECORD, Rotterdam Study, Turin Study. Hierfür wurde eine Erhebung zu Strategien und Programmen zur Förderung mentaler Gesundheit im Alter in den dazugehörigen Städten Amsterdam, Eindhoven, Hamburg, Helsinki, Kaunas, Krakau, London, Nord-Trøndelag, Paris, Prag, Rotterdam und Turin durchgeführt.

**Zielsetzung:**

Die detaillierte Bestandsaufnahme zu Gesundheitsstrategien und Programmen in diesen Städten diente der Untersuchung der Variabilität sowie der Ableitung von Empfehlungen für Wissenschaft, Politik und kommunale Planung unter Nutzung der Erkenntnisse der MINDMAP Städte.

**Materialien und Methoden:**

Die MINDMAP Partner benannten jeweils Experten mit bestmöglichem Überblick über das lokale Gesundheitsgeschehen (mentale Gesundheit) in den 12 Städten. Nach einem Prätest wurden semi-strukturierte Telefon-Interviews (1–2 h) durch dieselbe Person durchgeführt. Für die strukturierte Evaluation wurden das geriatrische Kontinuum der Funktionalität und der WHO Public-Health Framework for Healthy Ageing zugrunde gelegt.

**Ergebnisse:**

In der Vollerhebung (12/12 Städte) wurden 41 Strategien und 280 Programme auf Stadtebene berichtet. Die Evaluation ergab, dass ältere Personen und spezifische Zielgruppen sowie multidimensionale Programme stärkere Berücksichtigung finden könnten.

**Diskussion:**

Die Ergebnisse zeigen eine große Bandbreite im Umgang mit den Herausforderungen des gesunden Älterwerdens und unterstreichen physische und mentale Fähigkeiten älterer Menschen sowie deren Wechselwirkungen mit dem urbanen Lebensraum.

## Background and objectives

The World Health Organization (WHO) definition of healthy ageing is “the process of developing and maintaining the functional ability that enables well-being in older age” [1]. Hereby, functional ability in old age is assessed by physical and mental capacities, as well as individuals’ interactions with the environment [2]. Environmental factors include aspects defining the context of an individual person (contextual factors) and include health promotion, prevention and care policies and services, the environmental support by families and social networks as well as the built environment across the life-course [1]. From an individual’s point of view, the concept of person-environment fit is important as it includes individual health characteristics and capacity, societal needs and resources, interactive relationships of the older person and the environment including changes in people and places over time [1, p. 30].

From the geriatric perspective, this reflects the WHO International Classification of Functioning, Disability and Health (ICF) model [3]. The biopsychosocial model includes a coherent view of different biological, individual and social perspectives of health by integrating the concepts of body function and structure, activity, participation, medical and social aspects. Functional health including mental health, in particular, is seen as the outcome of interacting health conditions (e.g. diseases, disorders and injuries) and contextual factors. The latter comprise external environmental factors, such as social attitudes, architectural characteristics, legal and social structures and personal factors, such as gender, age, coping style, social background, education and behavioural patterns.

Understanding of the mental dimension of ageing requires consideration of the complex relationship between mental and physical geriatric syndromes (e.g. immobility, falls, incontinence, malnutrition, cognitive deficits, depression, anxiety and chronic pain) and its consequences for functional competence, impairments, disabilities and handicaps [4, 5]. In fact, the development of the “superordinate geriatric syndrome” frailty is seen as the “most problematic expression of population ageing” [6]. The frailty syndrome is characterised by progressive decline of physical and mental functions hindering the maintenance of independence, participation and autonomy [7]. Its clinical conditions caused by age-related decline in several physiological systems do increase the vulnerability in older age [8, 9]. Early disturbances in mental and/or cognitive function do influence physical functioning and mobility, a prerequisite of independence, and physical frailty predicts onset and course of late-life depression.

In addition, the development of disability and dependency in older persons through the dynamic frailty process is closely influenced by mental health problems [10]. Disability (e.g. impairments, activity limitations and participation restrictions) causes loss of functional competence and autonomy, increased vulnerability, and need of nursing care [11] with high costs [12]. In Germany, for example, disturbances in cognitive and mood contribute to the three predominant causes of nursing care. Even early impairments of higher mental/cognitive functioning may disturb physical function and mobility, in particular [13–15]. Vice versa, restrictions in physical activities can impair mental health well-being [16, 17]. Furthermore, recent evidence highlights the role of the urban environment in preventing the onset of functional decline (robustness – prefrailty – frailty – disability – nursing care – death). The risk of mental disorders has been shown to be higher in persons living in urban compared to rural areas [18]. The combination of urbanisation and ageing has relevant implications for public mental healthcare planning. These findings necessitate measures to prevent mental health disorders in order to counteract frailty in older age. Therefore, the maintenance of functional ability including mental health has a high priority in urban public healthcare planning policies and strategies, as well as public health intervention programmes to promote mental well-being and to prevent mental disorders in old age addressing these complex interrelationships [1].

**Fig. 1:**
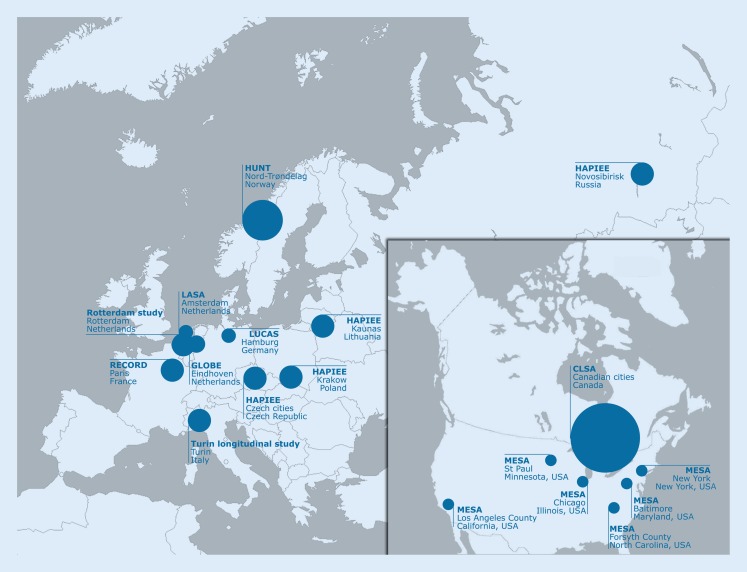
The MINDMAP cohorts and cities. Participating MINDMAP European cohorts and cities: Amsterdam (*LASA* Longitudinal Aging Study Amsterdam, LASA), Eindhoven (*GLOBE* GLOBE Study), Hamburg (*LUCAS* Longitudinal Urban Cohort Ageing Study), Krakow, Kaunas, Prague, Novosibirsk (*HAPIEE* Health, Alcohol and Psychosocial factors In Eastern Europe Study), Nord-Trøndelag (*HUNT* Nord-Trøndelag Health Study), Paris (*RECORD* Residential Environment and CORonary heart Disease Study), Rotterdam (Rotterdam Study), Turin (Turin Longitudinal Study) as well as London and Helsinki (both without a longitudinal urban cohort). Participating cohorts from the US and Canada: *MESA* Multi-Ethnic Study of Atherosclerosis and *CLSA* Canadian Longitudinal Study on Aging

Addressing these challenges, the European Union is currently funding (Horizon 2020 programme) the MINDMAP project “Promoting mental well-being and healthy ageing” (2016–2019) to identify opportunities offered by the urban environment for the promotion of mental well-being and cognitive function in older individuals. The MINDMAP project stresses the importance of early detection of preclinical stages of frailty, including mental components of functional competence. This project will advance understanding by bringing together longitudinal ageing cohort studies across European cities, the USA and Canada (Fig. 1). Analyses across these cohorts are planned by creating a harmonised data platform. This will facilitate to unravel pathways and multilevel interactions between the urban environment and social, behavioural, psychosocial and biological determinants of mental health and cognitive function in older adults. The project will examine the causes of variation in mental well-being and disorders in old age, both within and also between cities and identify national and urban policies for prevention and early diagnosis of mental disorders. The knowledge will significantly contribute to establish preventive strategies in urban settings to promote the mental dimension of healthy ageing to reduce its negative impact on mental well-being and comorbidities, and to preserve cognitive function in old age (for details http://www.mindmap-cities.eu/).

The MINDMAP partner Albertinen-Haus, Centre of Geriatrics and Gerontology, Hamburg contributes (a) with the Longitudinal Urban Cohort Ageing Study (LUCAS) [19] and (b) MINDMAP work package 9 “Geriatric perspective on promoting mental well-being and healthy ageing in cities”. The geriatric expertise enables the linkage between the urban environment and the older person’s functioning continuum in order to identify target groups for interventions by addressing mental health and cognitive function as relevant impact on the frailty process [20].

For a better understanding and profound data interpretation across the European MINDMAP longitudinal ageing cohorts (harmonised data platform), the work package 9 first milestone was a survey of healthcare planning policies, strategies and programmes addressing the heterogeneous older population by covering mental health promotion, mental disorder prevention, treatment, recovery, rehabilitation and nursing care.

Objectives of this MINDMAP survey:

- To collect detailed information on healthcare planning policies, strategies and programmes across the European MINDMAP consortium cities with longitudinal cohorts.
- To analyse how healthcare planning policies and strategies in European MINDMAP cities actually consider behavioural, psychosocial, physical urban environmental, geriatric medical, national and local political determinants of mental health with respect to promote mental well-being and healthy ageing.
- To examine variations about how healthcare systems deal with the consequences of mental impairment for physical function across the European MINDMAP cities.
- To delineate recommendations for policies and planners using the experiences from evidence-based practice feedback from the European MINDMAP cities.

## Material and methods

First, the WHO “Mental Health Policy and Practice across Europe” [21] was consulted together with the principal MINDMAP project coordinator (work package 1) and the work package leaders responsible for the analyses of the impact of mental health policies on the mental dimension of healthy ageing (work package 10), and this survey on healthcare planning policies, strategies and programmes addressing mental health promotion and mental disorder prevention for older people in the European MINDMAP cities (work package 9). Unfortunately, less attention has been given to mental health problems in older age than to working age adults [21].

Second, there was agreement to further search in regularly updated and accessible statistics and reports, such as the European profile of promotion and prevention of mental health [22], the WHO World Health Statistics [23], the WHO Mindbank report “More Inclusiveness Needed in Disability & Development” [24] and the Organisation for Economic Co-operation and Development (OECD) Health Statistics [25]. However, these sources had a lack of specific information on mental health promotion and mental disorder prevention for older people, neither on national, regional nor city levels. Therefore, we developed a comprehensive and structured survey on mental healthcare planning policies, strategies and programmes focussed on the city level (regional perspective). This was essential to collect relevant information from the 12 European MINDMAP cities of which the majority have access to a longitudinal ageing cohort in their cities (10 out of 12) or are domicile cities of additional MINDMAP work package partners (2 out of 12).

### Survey design and content

The following theoretical constructs were considered in the survey design: (a) the public health perspective, such as the WHO “Mental Health Action Plan” [11], the WHO “Global age-friendly cities” handbook [26], (b) in behavioural sciences, such as the salutogenesis concept [27], the model to promote social equity in health [28], and (c) the geriatric perspective for understanding frailty as a spectrum of conditions within the geriatric functional continuum [29, 30].

**Fig. 2:**
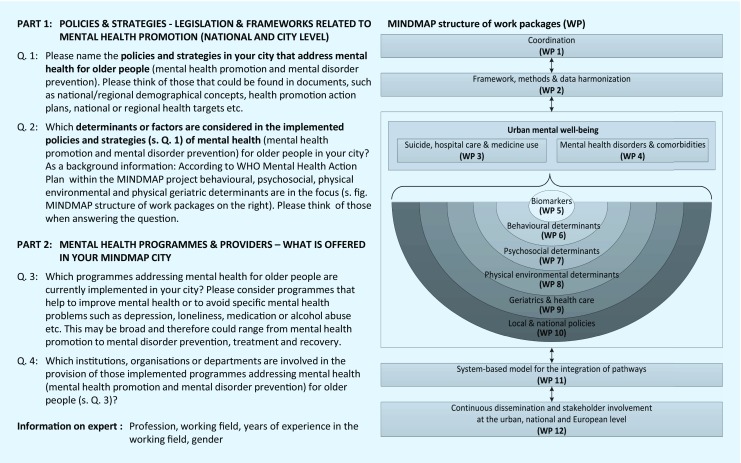
Structure of the MINDMAP survey and linkage to MINDMAP work packages. *WP* work packages

These recommendations and strategies resulted in a survey questionnaire divided into two parts. Part 1 addressed policies and strategies related to mental health promotion and mental disorder prevention on national and city levels according to the structure of the 12 work packages addressed in the interdisciplinary MINDMAP consortium (Fig. 2, questions part 1). Part 2 focussed on mental health programmes and providers. Extremely helpful for the actual survey design was the national questionnaire “Inventory taking of health promotion and prevention – offers and accessibility for older people in the community” edited by the Federal Centre for Health Education in Germany [31]. Fig. 2 also gives a rough overview of evaluating the broad variety of programmes and providers in each MINDMAP city (questions part 2).

### Pretesting

The questionnaire was pretested by public health experts from one city in Germany (member of the German Healthy City Network) and in Denmark. After the pretesting, the experts clearly recommended performing the survey as by a semi-structured telephone interview instead by using a written questionnaire. Therefore, the questionnaire layout was structured into a) summarising tables for each question supporting the interviewer to navigate through the semi-structured interview, and b) a written outline about the survey topics to help the interviewee to prepare the information to be retrieved in the semi-structured telephone interview.

### Survey performance

There was agreement to perform a comprehensive and complete (12 out of 12) survey in all European MINDMAP cities (in alphabetical order, geographic regions see Fig. 1):

1. Amsterdam (LASA: Longitudinal Aging Study Amsterdam, LASA),
2. Eindhoven (GLOBE: GLOBE Study),
3. Hamburg (LUCAS: Longitudinal Urban Cohort Ageing Study),
4. Helsinki (domicile city of MINDMAP work package 3),
5. Kaunas (HAPIEE: Health, Alcohol and Psychosocial factors In Eastern Europe Study),
6. Krakow (HAPIEE: Health, Alcohol and Psychosocial factors In Eastern Europe Study),
7. London (domicile city of MINDMAP work package 10),
8. Nord-Trøndelag (HUNT: Nord-Trøndelag Health Study),
9. Paris (RECORD: Residential Environment and CORonary heart Disease Study),
10. Prague (HAPIEE: Health, Alcohol and Psychosocial factors In Eastern Europe Study),
11. Rotterdam (Rotterdam Study) and
12. Turin (Turin Longitudinal Study).

All European MINDMAP consortium partners responsible for their 12 cities were invited to identify the most suitable expert in their city with an overview on the current mental health sector, and broad knowledge of healthcare planning policies, strategies and programmes in their city. We emphasised to consider the older population’s heterogenity as expressed as robust or non-robust (prefrail/frail) but predominantly not disabled. Therefore, depending on the administrative city structure, the MINDMAP partners searched for senior policy makers (persons working for the head of the city/regional health department or social affairs), representatives in a public health institute or a mental health care umbrella organisation/network. The MINDMAP partners provided the contact details of the experts (name, institution, e‑mail address, telephone extension) to the Albertinen-Haus (survey leader, work package 9).

Information about the MINDMAP consortium and the survey was distributed to each interview partner (interviewee), including an arrangement for a telephone appointment from the Albertinen-Haus. After interview acceptance, the interviewee received the written outline about the survey topics in order to support the interviewee in preparing the information according to the semi-structured telephone interview. All interviews (1–2 h/city) were in the English language, tape recorded and transcribed by the same PhD student (WJ) from the Albertinen-Haus from November 2016 to May 2017. In a few cases, two interviews took place in a single MINDMAP city if the first interviewee had referred.

### Survey evaluation

To assure high quality evaluation, the analysis of the transcribed interviews was performed in a structured way. For this purpose, the summarising tables for each question which helped the interviewer to navigate through the semi-structured interview were used as evaluation matrix. Two independent researchers (WJ and LN, PhD students) worked on each transcript while extracting and transforming the information given according to this evaluation matrix. Also, national and local strategy papers and local programmes referred to by the interviewees were intensively studied for this evaluation process. Then, the results of both the independent evaluations were collected and cross-checked. In cases of unequal assessment, a third researcher (UD, PhD) was involved to reach concordance. Finally, all interview transformation results as summarised in tables were submitted to the corresponding MINDMAP city interviewee to assure that all information given in the interview was correctly understood and reported. All interviewees from the 12 European MINDMAP cities gave their approval before all survey results were compared. Then, mainly descriptive methods were used for the comparison (e.g. absolute numbers, percentages and means), and all data were recorded in a data base, analysed and visualised using MS Excel 2010.

## Results

The collection of detailed information on mental healthcare planning policies, strategies and programmes from all 12 European MINDMAP cities was complete. In Krakow, London and the Nord-Trøndelag region, two interviews were performed with different experts. The interviewees’ professional background was mainly in the field of healthcare, most often medicine followed by psychiatry, psychology, nursing, public health and epidemiology (12 out of 15). Most interviewees worked at city councils (department of health or social affairs) or public health institutions (9 out of 15), the other experts in healthcare institutions or universities (6 out of 15) (Table 1).

**Table 1:** Characteristics of the experts in the MINDMAP survey (*n* = 15 experts in 12 European MINDMAP cities)

European MINDMAP cohort/city, country	Amsterdam, Netherlands	Eindhoven, Netherlands	Hamburg, Germany	Helsinki, Finland	Kaunas, Lithuania	Krakow, Poland		London, UK		Nord-Trøndelag Region, Norway		Paris, France	Prague, Czech Republic	Rotterdam, Netherlands	Turin, Italy
*Longitudinal cohort*	LASA	GLOBE	LUCAS	No cohort	HAPIEE	HAPIEE		No cohort		HUNT		RECORD	HAPIEE	Rotterdam Study	Turin Longitudinal Study
*No. interviews performed*	1	1	1	1	1	2		2		2		1	1	1	1
*Profession of expert*	Epidemiology	Social work	Psychology	Nursing science	Public health	Medicine	Epidemiology	Medicine	Social work	Medicine	Medicine	Public relations	Medicine	Public health advisory	Clinical psychiatry
*Working field of expert*	Public Health Service, Amsterdam	Public Health Service and Political Consulting	City of Hamburg, Ministry of Health, Department of Health Data and Health Promotion	City of Helsinki, Healthcare, Department of Senior Planning	City of Kaunas, Public Health Service	University Krakow, Public Health	University Krakow, Public Health	Public Health Organisation	University London, Community Care Service	Public Health Service	Hospital, Department for Mental Health and Addiction	City of Paris, Department of Social Affairs, Children and Health	National Ministry of Health, Department of Health Promotion, Public Health	Public Health Service, Rotterdam	Community Health Centre Turin, Mental Health Service
*Experience in the working field*	>10 years	3–10 years	>10 years	>10 years	0–2 years	>10 years	>10 years	>10 years	>10 years	0–2 years	>10 years	>10 years	>10 years	>10 years	>10 years
*Gender of expert*	Male	Female	Female	Female	Female	Male	Male	Male	Female	Female	Female	Male	Female	Male	Male

### Policies and strategies

Each city referred to at least one (1–7) policy and strategy on the city level but the experts also mentioned national policies and strategies. All addressed the range from mental health promotion, mental disorder prevention, treatment, recovery and care (Table 2, 3, 4 and 5). Out of 41 policies and strategies 32 identified at city level explicitly had older persons as a target group (older population) or senior citizens as a subgroup. The other nine were on general public health issues for all citizens. The older population was included in each policy/strategy on the city level in Hamburg (7 out of 7), Nord-Trøndelag (5 out of 5), Turin (3 out of 3), Helsinki (2 out of 2) and Eindhoven (1 out of 1) (Table 2, 3, 4 and 5). If the city level put minor emphasis on the older population (e. g. London and Kaunas) healthy ageing was covered on the national level instead. Mental health promotion and prevention was covered by 8 out of 41 policies/strategies (e. g. Hamburg “Pact for Prevention”), mental health diseases (22 out of 41) as by action plans on loneliness, depression or dementia (e. g. Prague “Professional Dementia Strategy”) (Table 2, 3, 4 and 5). More than half of the policies/strategies had a general targeting approach including aspects of health promotion, health prevention, treatment, recovery, rehabilitation and care but did not differentiate within the heterogeneous groups of senior citizens. However, six strategies explicitly focussed on mental health promotion and prevention (e. g. Amsterdam “Action Plan for the Prevention of Anxiety and Depression”) including early detection of risk groups, for example, older people living alone at home (Table 2, 3, 4 and 5).

**Table 2:** Overview of identified mental healthcare planning policies and strategies including addressed determinants in the 12 European MINDMAP cities (1/4)

	Overall		Amsterdam		Eindhoven		Hamburg	
Policy/strategy level	City	National	City	National	City	National	City	National
No. of policies/strategies identified	41	43	5	2	1	2	7	4
Name of the policies/strategies	s. each MINDMAP city	s. each MINDMAP city	– WHO Age Friendly City – GGD Health Monitoring Survey – Local Public Health Policy – Amsterdam Dementia Programme – Action Plan for the Prevention of Anxiety and Depression	– National Public Health Act – National Public Health Policy	– Eindhoven Loneliness Strategy	– National Public Health Act – National Public Health Policy	– Concept for Demography Hamburg – 1st Health Monitoring Survey for Older People – 2nd Health Monitoring Survey for Older People (LUCAS) – Federal State Law on Prevention Hamburg – Pact for Prevention Hamburg – Dementia Strategy Hamburg – Law on Participation of Older People	– National Demography Strategy – National Health Targets – Federal Law on Prevention – Local Alliances for people with dementia
Older population explicitly considered	32/41	30/43	4/5	0/2	1/1	0/2	7/7	4/4
Mental health promotion and prevention considered	8/41	14/43	0/5	0/2	0/1	0/2	1/7	1/4
Mental health diseases considered	22/41	24/43	3/5	0/2	1/1	0/2	6/7	3/4
Universal target group approach (including health promotion, prevention, treatment, recovery, rehabilitation and care)	22/41	21/43	2/5	2/2	0/1	2/2	4/7	3/4
Target groups explicitly: health promotion, prevention	6/41	5/43	1/5	0/2	0/1	0/2	2/7	0/4
Behavioural determinants (WP 6) considered	26/41	27/43	2/5	2/2	0/1	2/2	6/7	4/4
Psychosocial determinants (WP 7) considered	27/41	30/43	3/5	0/2	1/1	0/2	7/7	4/4
Physical urban environmental determinants (WP 8) considered	25/41	23/43	1/5	1/2	0/1	1/2	5/7	1/4
Physical, medical factors (diseases) (WP 9) considered	28/41	32/43	4/5	2/2	0/1	2/2	6/7	3/4
Determinants of local and national policies (WP 10) considered	32/41	40/43	3/5	2/2	0/1	2/2	7/7	4/4

**Table 3:** Overview of identified mental healthcare planning policies and strategies including addressed determinants in the 12 European MINDMAP cities (2/4)

	Helsinki		Kaunas		Krakow		London	
Policy/strategy level	City	National	City	National	City	National	City	National
No. of policies/strategies identified	2	3	1	3	4	5	2	8
Name of the policies/strategies	– The City of Helsinki Strategy Plan – Action Plan of Older people’s Health and Well-Being	– Law on Older Peoples Rights and Well-Being – National Strategy for Physical Activity, Promoting Health and Mental Well-Being – Act on Support of Caregivers	– WHO Healthy Cities Network	– Assurance of Healthy Ageing in Lithuania – National Public Health Care Development Programme – Mental Health Strategy and Suicide Prevention Action Plan	– Malopolska Public Health Strategy^a^ – Krakow Mental Health Strategy – WHO Healthy Cities Network – Krakow’s Pact for Seniors	– National Health Programme – Evaluation Report of the Implementation of the Tasks of the National Programme Mental Health – National Programme for Mental Health – National Retirement Strategy – National Law on Environmental Protection	– WHO Age Friendly Cities, Borough of Camden – WHO Healthy Cities Network	– Care Act – Equality Act – National Carers Strategy – Better Health Outcomes by 2020 – Mental Health Task Force – Preventing Suicide in England – Prime Minister’s Challenge on Dementia – Strategy for Healthy Ageing (in preparation)
Older population explicitly considered	2/2	3/3	0/1	3/3	2/4	2/5	1/2	7/8
Mental health promotion and prevention considered	0/2	2/3	0/1	3/3	2/4	1/5	0/2	1/8
Mental health diseases considered	1/2	1/3	0/1	3/3	2/4	3/5	1/2	5/8
Universal target group approach (including health promotion, prevention, treatment, recovery, rehabilitation and care)	2/2	2/3	0/1	1/3	1/4	1/5	2/2	3/8
Target groups explicitly: health promotion, prevention	0/2	0/3	1/1	0/3	0/4	2/5	0/2	1/8
Behavioural determinants (WP 6) considered	2/2	3/3	0/1	2/3	2/4	1/5	2/2	5/8
Psychosocial determinants (WP 7) considered	2/2	2/3	0/1	3/3	2/4	3/5	1/2	7/8
Physical urban environmental determinants (WP 8) considered	2/2	3/3	0/1	2/3	1/4	4/5	2/2	3/8
Physical, medical factors (diseases) (WP 9) considered	2/2	2/3	0/1	2/3	1/4	3/5	2/2	5/8
Determinants of local and national policies (WP 10) considered	2/2	3/3	1/1	2/3	2/4	3/5	2/2	8/8

^a^This strategy addresses the Malopolska region in Poland

**Table 4:** Overview of identified mental health care planning policies and strategies including addressed determinants in the 12 European MINDMAP cities (3/4)

	Nord-Trøndelag Region		Paris		Prague	
Policy/strategy level	Municipality	National	City	National	City	National
No. of policies/strategies identified	5	4	2	4	3	5
Name of the policies/strategies	– Vision: Quality of Life and Growth – General Health Plan: Coping for all (Levanger municipality) – General Health Plan: People should have what they need (Verdal municipality) – Plan for Care Services: Nursing and Care (Levanger municipality) – Plan for Care services: Nursing and Care (Verdal municipality)	– Public Health Act – Government Action Plan for the Implementation of the Health and Care 21 Strategy – National Report on Ageing – Norwegian Dementia Strategy	– Strategy on Ageing – Strategy on Environmental Health	– National Law on the Adaption to the Ageing of the Population – National Health Strategy – National Plan for Alzheimer and Related Diseases – French Neurodegenerative Diseases Plan	– WHO Healthy Cities Network – Grant for Healthy Ageing Projects – Professional Dementia Strategy	– Act on Public Health – Act on Health – Nation Public Health Strategy: Health 2020 – Mental Health Action Plan – National Action Plan for Positive Health
Older population explicitly considered	5/5	3/4	1/2	4/4	2/3	3/5
Mental health promotion and prevention considered	2/5	4/4	0/2	0/4	0/3	2/5
Mental health diseases considered	3/5	3/4	1/2	3/4	2/3	3/5
Universal target group approach (including health promotion, prevention, treatment, recovery, rehabilitation and care)	5/5	2/4	1/2	1/4	1/3	2/5
Target groups explicitly: health promotion, prevention	0/5	0/4	0/2	0/4	0/3	1/5
Behavioural determinants (WP 6) considered	4/5	2/4	1/2	1/4	1/3	3/5
Psychosocial determinants (WP 7) considered	5/5	3/4	1/2	2/4	1/3	4/5
Physical urban environmental determinants (WP 8) considered	5/5	3/4	2/2	1/4	1/3	3/5
Physical, medical factors (diseases) (WP 9) considered	4/5	3/4	1/2	4/4	1/3	4/5
Determinants of local and national policies (WP 10) considered	5/5	4/4	1/2	4/4	1/3	5/5

**Table 5:** Overview of identified mental health care planning policies and strategies including addressed determinants in the 12 European MINDMAP cities (4/4)

	Rotterdam		Turin	
Policy/strategy level	City	National	City	National
No. of policies/strategies identified	6	2	3	1
Name of the policies/strategies	– WHO Healthy Cities Network – GGD Health Monitoring – Rotterdam Vital City – Combat Loneliness Strategy – To Live Long at Home – People Make the Inner City	– National Public Health Act – National Mental Health Programme	– Law to Promote Health Activities for Older People – WHO European Healthy Cities Network – Home Support Plan	– National Health Recommendations
Older population explicitly considered	4/6	0/2	3/3	1/1
Mental health promotion and prevention considered	3/6	0/2	0/3	0/1
Mental health diseases considered	2/6	0/2	0/3	0/1
Universal target group approach (including health promotion, prevention, treatment, recovery, rehabilitation and care)	2/6	1/2	2/3	1/1
Target groups explicitly: health promotion, prevention	2/6	1/2	0/3	0/1
Behavioural determinants (WP 6) considered	5/6	2/2	1/3	0/1
Psychosocial determinants (WP 7) considered	4/6	2/2	0/3	0/1
Physical urban environmental determinants (WP 8) considered	5/6	1/2	1/3	0/1
Physical, medical factors (diseases) (WP 9) considered	4/6	1/2	3/3	1/1
Determinants of local and national policies (WP 10) considered	6//6	2/2	2/3	1/1

According to the MINDMAP consortium structure WP 6–WP 10 (Fig. 2) mental health on the city level was categorised into behavioural (26/41), psychosocial (27/41), physical urban environmental (25/41), geriatric medical determinants (28/41), and linked to local and/or national policies and strategies (32/41). In Helsinki, explicitly all five determinants were part of each policy and strategy. For example, its “Action Plan of Older People’s Health and Well-being” addresses (1) care services, (2) living and housing conditions, (3) public transportation and physical well-being, (4) equal rights for people with memory problems, (5) information and knowledge on healthy ageing and (6) referring to additional policies on city and national level. In Amsterdam, Hamburg, Krakow, London, Nord-Trøndelag, Paris and Rotterdam, at least three of these determinants were addressed in the context of half or more of the policies (Table 2, 3, 4 and 5).

### Programmes

A total of 280 programmes to promote mental well-being, prevent mental disorders or provide mental care were reported of which 197 were exclusively for the older population. Others included older age in cross-generational programmes. There were 91/280 programmes (32.5%) explicitly dedicated to health promotion and prevention for older people, in particular (e. g. Turin: outdoor physical activity group exercise at the river Po waterfront; London: “Fit as a Fiddle” focussing either on cognitive training using tai-chi, physical activity or social participation). Programmes for selected groups, mainly diagnosis-based, were also offered (128/280, 45.7%), e. g. Nord-Trøndelag region: dementia teams performing case management including home visits and Eindhoven: specific architectural design of nursing homes for patients who suffer from dementia. Nearly no programme had predefined indicators for future outcome measurements.

**Fig. 3:**
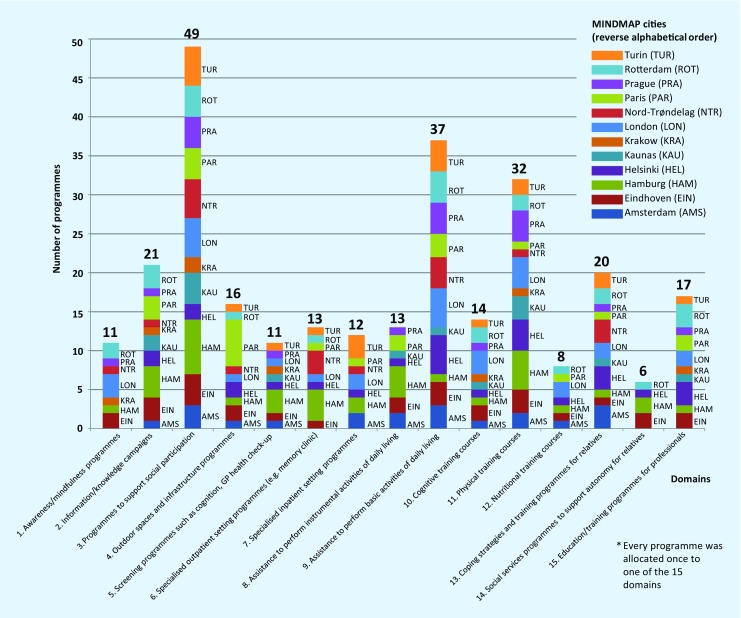
Overview of mental health programmes in old age in the 12 MINDMAP cities (*n* = 280 programmes*). Explanation of the programme domains: *1.* awareness/mindfulness programmes (i. e. social isolation, loneliness, bereavement groups, retirement transition). *2.* Information/knowledge campaigns, such as public lectures, awareness week/open house, brochure e. g. addressing substance abuse, depression, dementia. *3.* Programmes to support active social participation via affordability, availability, accessibility, acceptability (i. e. volunteering options, neighborhood participation/shared housing initiative, senior centres, lunch specials). *4.* Outdoor spaces and infrastructure programmes (i. e. accessible buildings, well-maintained pavements, good street lighting, public toilets, green spaces, public transport). *5.* Screening programmes: cognition, general practitioner (GP) health check-up, sensory system (e.g. vision, hearing, functional status, diabetes, heart diseases, medication review; dental check-up). *6.* Specialised outpatient setting programmes: memory clinic including geriatric, neuropsychological and gerontopsychiatric expertise (e.g. dementia diagnostics, coping with critical and traumatic life events), other outpatient interventions (e.g. preventive home visits, fall clinic). *7.* Specialised inpatient setting programmes, such as psychiatric intensive care units or hospital wards for cognitively impaired geriatric patients. *8.* Assistance to perform instrumental activities of daily living (e.g. home helpers, financial advice, meals on wheels, carpools). *9.* Assistance to perform basic activities of daily living (e.g. home-based nursing care, day care centre). *10.* Cognitive training courses (e.g. professional mental stimulation by neuropsychologist, “brain walking”, tai-chi, choir, painting classes). *11.* Physical training courses (e.g. muscle training, balance, endurance, fall prevention). *12.* Nutritional training courses (e.g. cooking classes, intervention/awareness of malnutrition in old age). *13.* Coping strategies and training programmes for relatives (e.g. family or caregiver training, crisis intervention). *14.* Social services programmes to support autonomy for relatives (e.g. counselling to get help with dementia, home care, equipment adaptation). *15.* Education/information/training programmes for professionals (e.g. public lectures, general practitioner quality circle)

Each of the 280 city programmes was allocated by the interviewees to one of the 15 domains, depending on its main intervention issue (Fig. 3). For example, the 12 cities provided 49 programmes (1–7 programmes/city) to support social participation (e. g. Paris: senior ambassadors to overcome social isolation by bringing two older persons together; senior centers in 8/12 cities where older persons can meet) (Fig. 3, domain 3).

## Discussion

### Policies and strategies

All European MINDMAP cities have policies and strategies addressing the older population, ranging from 1–7 policies/strategies on city level. However, this analysis revealed that merely half of the policies and strategies integrated at least three out of the five determinants specified by the MINDMAP consortium work packages WP 6–WP 10 (Fig. 2; Table 2, 3, 4 and 5). Mental health as an essential component of healthy ageing does require both physical and mental capacities for functional ability [1, 11]. Yet, this has not been appropriately addressed in the policies/strategies identified. The major focus of most policies was on diseases without also emphasising and strengthening old persons’ mental resources [27]. Both these aspects should be considered in healthcare planning policies and strategies [1].

Both the public health [26, 28] and geriatric perspectives do consider the relationship between physical syndromes, including frailty and mental health [29, 30]. Multiple determinants must consider interacting health conditions (diseases, disorders and injuries), external environmental and internal personal factors (ICF model) [3]. Life-course determinants as emphasised in the *Canadian Initiative on Frailty and Aging* could also be considered in the analyses addressed in MINDMAP WP 6–WP 10. Promotion of healthy ageing and disease prevention, and thereby, the preservation of physiological reserves do have an impact on frailty which is closely related to adverse outcome, morbidity, disability, hospitalisation, nursing care and death [30]. Therefore, components of the frailty process might also be on the agenda of the MINDMAP analyses (harmonised data platform), and in strategies and intervention programmes on the MINDMAP city level. An example of policies and interventions on the MINDMAP city level to be mentioned is the European Innovation Partnership on Active and Healthy Ageing platform “Innovation for age-friendly buildings, cities and environment” the MINDMAP cities Krakow and Hamburg are engaged in (https://ec.europa.eu/research/innovation-union/pdf/active-healthy-ageing/d4_action_plan.pdf; access: 05.06.2017). In Germany, a National Dementia Strategy is currently planned as part of the federal government’s demography strategy (http://www.allianz-fuer-demenz.defileadmin/de.allianz-fuer-demenz/content.de/downloads/GemeinsamfuerMenschenmitDemenz-Handlungsfelder-deutsch.pdf; access: 05.06.2017).

### Programmes

The heterogeneous spectrum of the 280 city programmes was allocated to the 15 domains addressed in the interviews (Fig. 3). Most common were mental disorder treatment programmes or care provision to support social participation (49 programmes, domain 3) or to assist with activities of daily living (37 programmes, domain 9), followed by information campaigns (21 programmes, domain 2) and training for relatives, caregivers (20 programmes, domain 13) and healthcare professionals (17 programmes, domain 15).

Combinations of interacting medical, functional, psychosocial and environmental factors contribute to the development of disability and dependency. Therefore, it is unlikely that a focus on single factors might lead to meaningful results. Instead, successful preventative models for older persons used a multidimensional approach [32]. Promotion of mental well-being and prevention of mental disorders in older persons were less common in the MINDMAP cities. There was a predominance of single-dimensional programmes, for example, physical training courses (32 programmes, domain 11). However, not all cities seemed to actually connect such programmes by using a multidimensional approach to outdoor spaces and infrastructure plans (16 programmes, domain 4), to courses of cognitive training (14 programmes, domain 10), awareness programmes (11 programmes, domain 1) or courses on nutrition (8 programmes, domain 12).

There were education or training programmes for healthcare professionals (domain 15) in 10 of the 12 cities. Screening instruments might be applied in such programmes to concentrate on appropriate older population subgroups, and for raising awareness of multifactorial interrelationships. As an example, the guide “Safe and sound” was developed for independent community-dwelling older people. Meanwhile, it is used in the context of an education programme in Hamburg to support professionals, e.g. GP practices, physiotherapist practices, sports clubs, social services, and community care information centers (*Pflegestützpunkt*) to screen for the risk of falling and to provide advice dedicated to improve stability and maintenance of mobility [33].

The value of existing policies/strategies and programmes offered is likely to be underestimated because indicators to evaluate effects of programmes offered to defined target groups were almost unavailable although 10 of the 12 MINDMAP cities might have access to multidimensional data from longitudinal cohort studies (Fig. 1). Thus, supplementary indices could have been used from screening or assessment in order to get information on programme acceptance and effects. For example, the “Municipal Masterplan 2015–2030 – Vision: Quality of life and growth” in the MINDMAP partner region Nord-Trøndelag does include predefined indicators for the strategies’ performance review:

- life expectancy,
- self-reported health and quality of life,
- years of life with good health,
- physical activity (bicycling and self-reported physical activity),
- participation in cultural activities/volunteering,
- traffic (number of passengers using public transport and car traffic counts).

Data from the HUNT cohort (HUNT4) are used for this purpose (http://www.levanger.kommune.no/Global/dokumenter/kommuneplan_samfunn_english.pdf, access: 05.06.2017). Another example is the application of the LUCAS functional ability index [20] as screening and as indicator to measure effects of a programme of transsectoral healthcare coordination for older independent community-dwelling people (programme in domain 6) (https://www.albertinen.de/krankenhaeuser/geriatrischeklinik/leistungsspektrum/netzwerkgesundaktiv; access: 05.06.2017).

The spectrum of policies, strategies and programmes collated in this European MINDMAP city survey will be a rich source for a better understanding of the diversity within and between the longitudinal cohorts of the MINDMAP project (harmonised metadata platform). Further research is planned in the MINDMAP project (2016–2020) to develop recommendations for policies and plans by using evidence-based practice feedback from the European MINDMAP cities with longitudinal ageing cohorts.

### Limitations and strengths

The information retrieved from the survey may be limited as only one (maximum 2) local expert, even a person with best available background knowledge in the field of public health was interviewed. Consequently, only those policies, strategies and programmes were taken into account which were reported by the local MINDMAP interviewees. However, all MINDMAP consortium partners carefully searched in their cities for experts and explained the survey details in the mother tongue. The consortium had decided that all interviews should be performed in English. However, there might still be an information bias. Therefore, a written outline of the interview topics was sent in advance to prepare the information, and feedback from the interviewee based on a written summarising interview synopsis was obtained. It should also be noticed that policies, strategies and programmes on national and city levels cannot be compared in total, and recommendations cannot be simply transferred from one to another city/metropole region, also because of different healthcare systems in Europe.

A strength of this survey is the complete data collection from each of the 12 European MINDMAP cities. Furthermore, the development, performance and analyses of this survey were carried out in a way that was as structured as possible (review of existing databases/reports, development of interview questions based on different adequate models, structured identification and preparation of the interviewee by provision of written outline in advance, semi-structured interview, transcription of each interview, structured analysis using predefined summarizing tables, and feedback based on a written summarising interview synopsis).

Further research is clearly needed bringing together national and city level policies and the translation and implementation into linking programmes relevant to practice. Also, appropriate indicators for outcome measurement should be developed and applied.

## Conclusion

The survey results presented here elaborate evidence of broad variety and creativity to cope with the challenges of ageing, and to address physical and mental capacities in older individuals and their dynamic interactions in an urban environment. Rich material on policies, strategies and programmes was found in each of the 12 European MINDMAP cities. There are some main conclusions from the analyses relevant for present and future developments and measures, respectively:

- National and city level policies/strategies should correspond with what is actually offered on the city level.
- Data sources from longitudinal ageing cohorts, central city registries or health registries could be used to appropriately define indicators for the measurement of programme effects.
- Available evidence-based gerontologic/geriatric expertise could be integrated in order to develop and to tailor multidimensional programmes for promotion of mental well-being and mental disorder prevention in the older population.
- Screening or assessment indices could be applied to address special (risk) groups within the growing heterogeneous older part of our populations.
