# Correction to: The MINDMAP project: mental well-being in urban environments: Design and first results of a survey on healthcare planning policies, strategies and programmes that address mental health promotion and mental disorder prevention for older people in Europe

**DOI:** 10.1007/s00391-017-1325-0

**Published:** 2017-10-04

**Authors:** L. Neumann, U. Dapp, W. Jacobsen, F. van Lenthe, W. von Renteln-Kruse

**Affiliations:** 1 Geriatrics Centre, Scientific Department at the University of Hamburg, Albertinen-Haus, Sellhopsweg 18–22, 22459 Hamburg, Germany; 2 Department of Public Health, Erasmus University Rotterdam, Rotterdam, The Netherlands

**Correction to:**

**Z Gerontol Geriat 2017**

10.1007/s00391-017-1290-7

The article “The MINDMAP project: mental well-being in urban environments. Design and first results of a survey on healthcare planning policies, strategies and programmes that address mental health promotion and mental disorder prevention for older people in Europe”, written by L. Neumann, U. Dapp, W. Jacobsen, F. van Lenthe, W. von Renteln-Kruse, was originally published electronically on the publisher’s internet portal (currently SpringerLink) on 17 August 2017 without open access.

With the author(s)’ decision to opt for Open Choice the copyright of the article changed on September 15^th^ 2017 to © The Author(s) [2017] and the article is forthwith distributed under the terms of the Creative Commons Attribution 4.0 International License (http://creativecommons.org/licenses/by/4.0/), which permits use, duplication, adaptation, distribution and reproduction in any medium or format, as long as you give appropriate credit to the original author(s) and the source, provide a link to the Creative Commons license and indicate if changes were made.

The original article was corrected.

